# Interaction of electrically evoked activity with intrinsic dynamics of cultured cortical networks with and without functional fast GABAergic synaptic transmission

**DOI:** 10.3389/fncel.2015.00272

**Published:** 2015-07-17

**Authors:** Thomas Baltz, Thomas Voigt

**Affiliations:** ^1^Institut für Physiologie, Medizinische Fakultät, Otto-von-Guericke-Universität Magdeburg, MagdeburgGermany; ^2^Center for Behavioral Brain Sciences, MagdeburgGermany

**Keywords:** MEA, multielectrode arrays, gamma-aminobutyric acid, neocortex, cerebral cortex, stimulation, network activity, cell culture

## Abstract

The modulation of neuronal activity by means of electrical stimulation is a successful therapeutic approach for patients suffering from a variety of central nervous system disorders. Prototypic networks formed by cultured cortical neurons represent an important model system to gain general insights in the input–output relationships of neuronal tissue. These networks undergo a multitude of developmental changes during their maturation, such as the excitatory–inhibitory shift of the neurotransmitter GABA. Very few studies have addressed how the output properties to a given stimulus change with ongoing development. Here, we investigate input–output relationships of cultured cortical networks by probing cultures with and without functional GABA_A_ergic synaptic transmission with a set of stimulation paradigms at various stages of maturation. On the cellular level, low stimulation rates (<15 Hz) led to reliable neuronal responses; higher rates were increasingly ineffective. Similarly, on the network level, lowest stimulation rates (<0.1 Hz) lead to maximal output rates at all ages, indicating a network wide refractory period after each stimulus. In cultures aged 3 weeks and older, a gradual recovery of the network excitability within tens of milliseconds was in contrast to an abrupt recovery after about 5 s in cultures with absent GABA_A_ergic synaptic transmission. In these GABA deficient cultures evoked responses were prolonged and had multiple discharges. Furthermore, the network excitability changed periodically, with a very slow spontaneous change of the overall network activity in the minute range, which was not observed in cultures with absent GABA_A_ergic synaptic transmission. The electrically evoked activity of cultured cortical networks, therefore, is governed by at least two potentially interacting mechanisms: A refractory period in the order of a few seconds and a very slow GABA dependent oscillation of the network excitability.

## Introduction

To date electrical stimulation of neuronal structures has become an important therapy for patients suffering from central nervous system disorders such as Parkinson’s disease, epilepsy and deafness [for review see for example Clark (2006), Kringelbach et al. (2007), Kipke et al. (2008), Moore and Shannon (2009), Lenarz et al. (2013)]. It has also been proposed to replace impaired or degenerated neuronal circuits in the visual system. For example the remaining neurons in the visual system can be excited by electrical pulses in patients suffering from blindness, which can restore or enhance visual percepts to a certain extend (Roessler et al., 2009; Walter, 2009; Ahuja et al., 2011; Klauke et al., 2011; Zrenner et al., 2011; Lewis et al., 2015). Intracellular electrical stimulation, i.e., the injection of currents into single neurons, has been used to characterize single neuron firing properties, such as regular or fast spiking (McCormick et al., 1985), bursting (Connors and Gutnick, 1990) or resonance phenomena (Hutcheon and Yarom, 2000; Izhikevich, 2006). Extracellular electrical stimulation, in contrast, acts on the network level by activating multiple elements in the network simultaneously, which can lead to complex responses due to extensive neuronal and synaptic interactions. This is of interest because these interactions lead to variable responses of the neuronal network to repeated representations of the very same stimulus, which in turn impedes experimental findings or might induce an undesired variability in percepts of patients. General insights into input–output relationships of neuronal networks are therefore important to allow precise predictions of the networks output in response to a given stimulus. Similarly, it would be of great advance if experimenters could deduce to certain network properties, such as the efficacy of GABAergic inhibition, on the basis of specific output characteristics.

Cortical neurons grown onto arrays of microelectrodes (MEAs) form networks with straightforward complexity and, therefore, represent a relative easy to understand model system. After neurons are born, they interconnect via synaptic contacts and spontaneously develop slow oscillatory synchronized activity, which is typical for developing networks and observed in various neuronal structures *in vivo* as well as *in vitro*, including the cerebral cortex, the hippocampus, the spinal cord, as well as in the developing retina (Robinson et al., 1993; Feller, 1999; O’Donovan, 1999; Garaschuk et al., 2000; Chiu and Weliky, 2001; Khazipov et al., 2001; Streit et al., 2001; Harris et al., 2002; Leinekugel et al., 2002; Khazipov et al., 2004; Van Pelt et al., 2004; Arnold et al., 2005; Wagenaar et al., 2005; Chiappalone et al., 2006; McCabe et al., 2006; Wagenaar et al., 2006a; Allene et al., 2008; Baltz et al., 2010, 2011). Phenomenologically, most of the electrical activity observed in developing structures of the central nervous system is confined in recurrent short bursts of action potentials, which are accompanied by a large increase of the intracellular calcium concentration. In cortical cultures, periodic bursting emerges at about the end of the first week *in vitro* (Wagenaar et al., 2006a; Baltz et al., 2011). With ongoing development, the initial stereotyped activity changes and more complex patterns evolve (Kamioka et al., 1996; Marom and Shahaf, 2002), in part due to maturation of the GABAergic system (Baltz et al., 2010).

Once generated, cortical neurons grown on MEAs allow non-invasive, long-term measurements of the spontaneous activity (Maeda et al., 1995; Morefield et al., 2000; Potter and DeMarse, 2001; Van Pelt et al., 2004; Wagenaar et al., 2005, 2006a; Baltz et al., 2010; Gramowski et al., 2010; Weir et al., 2014) and electrical stimulation of the same cells over extended periods of time (Wagenaar et al., 2006b; Brewer et al., 2009; Bologna et al., 2010; Ide et al., 2010; Weihberger et al., 2013; Keren and Marom, 2014). Consequently, such a preparation has a variety of applications as for example studying basic neuronal mechanisms such as information processing, neuronal plasticity, neurotoxicity screening, biocompatibility testing as well as understanding sources of human disorders (Shahaf and Marom, 2001; Eytan et al., 2003; Wagenaar et al., 2005; Bakkum et al., 2008; Kermany et al., 2010; Wallach and Marom, 2012; Dranias et al., 2013; Jantzen et al., 2013; Charkhkar et al., 2014; Frega et al., 2014; Gullo et al., 2014a,b; Hedrich et al., 2014; Mack et al., 2014).

When probing cultured cortical networks by means of extracellular electrical pulses typically an early and a late component of post stimulus spike responses can be distinguished (Jimbo et al., 2000; Marom and Shahaf, 2002; Wagenaar et al., 2004). The early component has latencies up to 20 ms and is thought to be mainly the result of direct antidromic axonal stimulation (Wagenaar et al., 2004). These responses are, therefore, independent of synaptic activity, occur with high reliability and a relatively low temporal jitter a few milliseconds after stimulation (Jimbo et al., 2000; Wagenaar et al., 2004). Early postsynaptic spikes can also occur a few milliseconds after stimulation, but they are characterized by a higher temporal jitter and a lower reliability (Wagenaar et al., 2004). In contrast to the early component of post stimulus spike responses, the late component is purely synaptically mediated and characterized by reverberating spike burst with highly variable latencies (Marom and Shahaf, 2002; Wagenaar et al., 2004).

In cortical cultures a variety of stimulation protocols have been used, for example, to control the oscillatory population activity as a putative treatment for epilepsy (Wagenaar et al., 2005), to study adaptation phenomena (Eytan et al., 2003; Wagenaar et al., 2006b) or neuronal plasticity (Jimbo et al., 1998, 1999; Tateno and Jimbo, 1999; Shahaf and Marom, 2001; Bakkum et al., 2008; Chiappalone et al., 2008; Stegenga et al., 2009; Bologna et al., 2010). The protocols used range from long-term low frequency (Wagenaar et al., 2006b; Vajda et al., 2008; Bologna et al., 2010) to high frequency ‘tetanic’ stimuli (Jimbo et al., 1998, 1999; Wagenaar et al., 2006b; Chiappalone et al., 2008).

Relatively little emphasis has been put to systematically investigate how the output properties in stimulation experiments change with respect to the actual state of the network, the frequency of applied stimuli and the developmental stage. Finding adequate and reliable stimulation parameters to evoke or induce a desired network output over the course of network maturation can be difficult (Wagenaar et al., 2006b) due to ongoing changes in synaptic coupling strength, the neuritic outgrowth and the differentiation of neuronal subtypes with its different firing properties (e.g., regular spiking, fast spiking, or bursting). In addition, the mode of action of important neurotransmitters, particularly of GABA, undergo developmental changes in many neuronal structures (Feller, 1999; Milner and Landmesser, 1999; O’Donovan, 1999; Ben-Ari et al., 2007), including cortical cultures (Baltz et al., 2010). All these developmental changes are expected to alter the input–output relationship of neuronal networks and the implications of these alterations may, if unknown, hamper the interpretation or reproducibility of findings in stimulation experiments.

In the present study input–output relationships of cultured cortical networks are systematically studied during the first 3 weeks of their development *in vitro*. We focus on developmental changes of the electrically evoked network activity in cultures with intact and with impaired fast GABAergic synaptic transmission to assess the contribution of the emerging inhibitory GABA action. Furthermore, we apply several protocols to characterize the general electrically evoked response of ‘mature’ networks (i.e., cultures older than 3 weeks *in vitro*).

## Materials and Methods

### Cell culture

All experimental procedures were approved by local government (Landesverwaltungsamt Halle, Germany, AZ 42502-3-616). Pregnant rats were euthanized by intraperitoneal injection of an overdose of choral hydrate (10% chloral hydrate, 1 ml/100 g body weight). Embryonic, as well as P0–P3 rats were killed by decapitation. For cultivation of cortical neurons plasma cleaned (Harrick Plasma, Ithaca, NY, USA) microelectrode array (MEA, Multi Channel Systems [MCS], Reutlingen, Germany) were treated overnight with poly-D-lysine (0.1 mg/ml in borate buffer, pH 8.5, 36°C). To suppress cell proliferation and to support neuronal survival (Schmalenbach and Müller, 1993; de Lima and Voigt, 1999) a feeder layer of purified astroglial cells was prepared from cerebral hemispheres of P0–P3 Sprague–Dawley rats as reported in detail previously (de Lima and Voigt, 1999). The astroglial cells were plated onto the MEA substrate with a density of 500 cells/mm^2^ 5 days before the neurons. Young neurons were prepared from cerebral cortices of embryonic Sprague–Dawley rats at embryonic day E16 (day after insemination was E1; birth = E22). The cortical tissue was obtained from the lateral parts of the telencephalic vesicles (excluding hippocampal and basal telencephalic anlagen). The cells were dissociated with trypsin/EDTA and seeded at a density of 1200 cells/mm^2^ onto the feeder layer. All cultures were maintained in N2 medium (75% DMEM, 25% Ham’s F12, and N2 supplement; Invitrogen, Carlsbad, CA, USA) in a humidified 5% CO_2_/95% air atmosphere at 36°C. The culture chamber was sealed by a screw cap to prevent infection and evaporation. Within the incubator, the cap was loosened to allow gas circulation. Some MEA cultures were raised and recorded in the presence of the specific GABA_A_R blocker gabazine (20 μM), added 3 h after plating. We favored gabazine for the chronic blockade, as in experiments where we acutely blocked GABA_A_Rs with bicuculline (5 μM) the network activity recovered from hyper synchronous activity to more clustered and asynchronous activity after washing out the substance, whereas washing out gabazine was more difficult in our hands. Furthermore, when we applied gabazine acutely for longer periods (>>1 h) with concentrations as low as 0.5 μM, the network activity remained stereotyped throughout the recording period similar to acute blockade with bicuculline. Thus, a breakdown of gabazine over the period of several days in chronic experiments seemed to be unlikely.

Once to twice a week medium was replenished with fresh N2 medium by changing half of the total medium volume, containing gabazine in case chronically blocked cultures [for details of culture techniques see (de Lima and Voigt, 1999; Baltz et al., 2010)].

### Drugs and Drug Application

All drugs were dissolved to 100–1000x stocks, stored at –20°C, and diluted to final concentration just before application. We purchased (-)-bicuculline methiodide (bicuculline) from RBI (RBI/Sigma, Deisenhofen, Germany), and D-2-amino-5-phosphonopentanoic acid (D-AP5), 6-imino-3-(4-methoxyphenyl)-1(6H)-pyridazinebutanoic acid hydrobromide (gabazine), and 6-cyano-7-nitro-quinoxaline-2,3-dione disodium (CNQX) from Tocris Cookson (Biotrend, Cologne, Germany). Drugs were applied directly from the stocks and cultures were allowed to equilibrate for at least 20 min before the recording starts to avoid a putative interference of transient changes in the network activity that might have been induced by culture handling (Wagenaar et al., 2006a).

### MEA Recordings and Data Processing

Recording of electrical activity was carried out using MEAs with 59 substrate-embedded titanium nitride recording electrodes, arranged in a 10 × 6 rectangular array with one electrode missing in the first column (MCS). The electrodes, 30 μm in diameter, had an inter-electrode distance (center to center) of 500 μm. Signals were amplified 1100× and sampled at 25 kHz using a preamplifier (MEA1060-Inv-BC) and data acquisition card (both MCS). The activity of individual cultures was monitored at 36°C using MC_Rack software (MCS). Recordings for different culture conditions were always age-matched. Spikes were detected on-line on the band-pass filtered (0.15–3.5 kHz) signal, using a threshold of –5× SD from background noise. Custom-written MATLAB (version 2007b, Mathworks, Natnick, MA, USA) programs were used for off-line analysis.

### Stimulation Protocols and Data Analysis of Electrically Evoked Activity

#### General Stimulation Properties

Charge-balanced, rectangular negative-first current pulses with a total duration of 400 μs and maximum amplitude of 25 μA were used. To minimize the detection of spurious spikes after stimulation the electrodes were kept disconnected for an additional 500 μs long period after each stimulation pulse to reduce the stimulation artifact by a blanking circuit in the amplifier. Additionally, supra-threshold peaks up to 2 ms post stimulus were discarded in the analysis. Some stimulation protocols were initially applied in preliminary experiments with an additional set of cultures; the obtained datasets were merged with the rest of the data.

#### Direct Responses to 1 to 100 Hz Stimulation

To investigate the frequency dependence of direct responses, one hundred 20 s long stimulation blocks with pulse frequency in the range from 1 to 100 Hz were applied through a single electrode in the presence of the glutamatergic and GABAergic synaptic blockers CNQX (50 μM), D-AP5 (50 μM), and bicuculline (10 μM). The stimulation blocks were pseudo randomized in order to avoid putative adaptation effects, and intermittent by 15 s long periods without stimulation. The choice of the stimulating electrode was based on the SNR of evoked spikes and the capability to evoke direct neuronal responses with low latency (<10 ms) in response to low-frequency (1 Hz) T-Pulses. Principal component analysis was performed on all spike waveforms detected on a given electrode and were projected onto the first two components. Data sets from stimulation-recording electrode pairs were neglected when multiple or overlapping clusters were observed. Furthermore, data sets were not considered in the analysis if neurons fired spontaneously in the presence of synaptic blockers.

**FIGURE 1:**
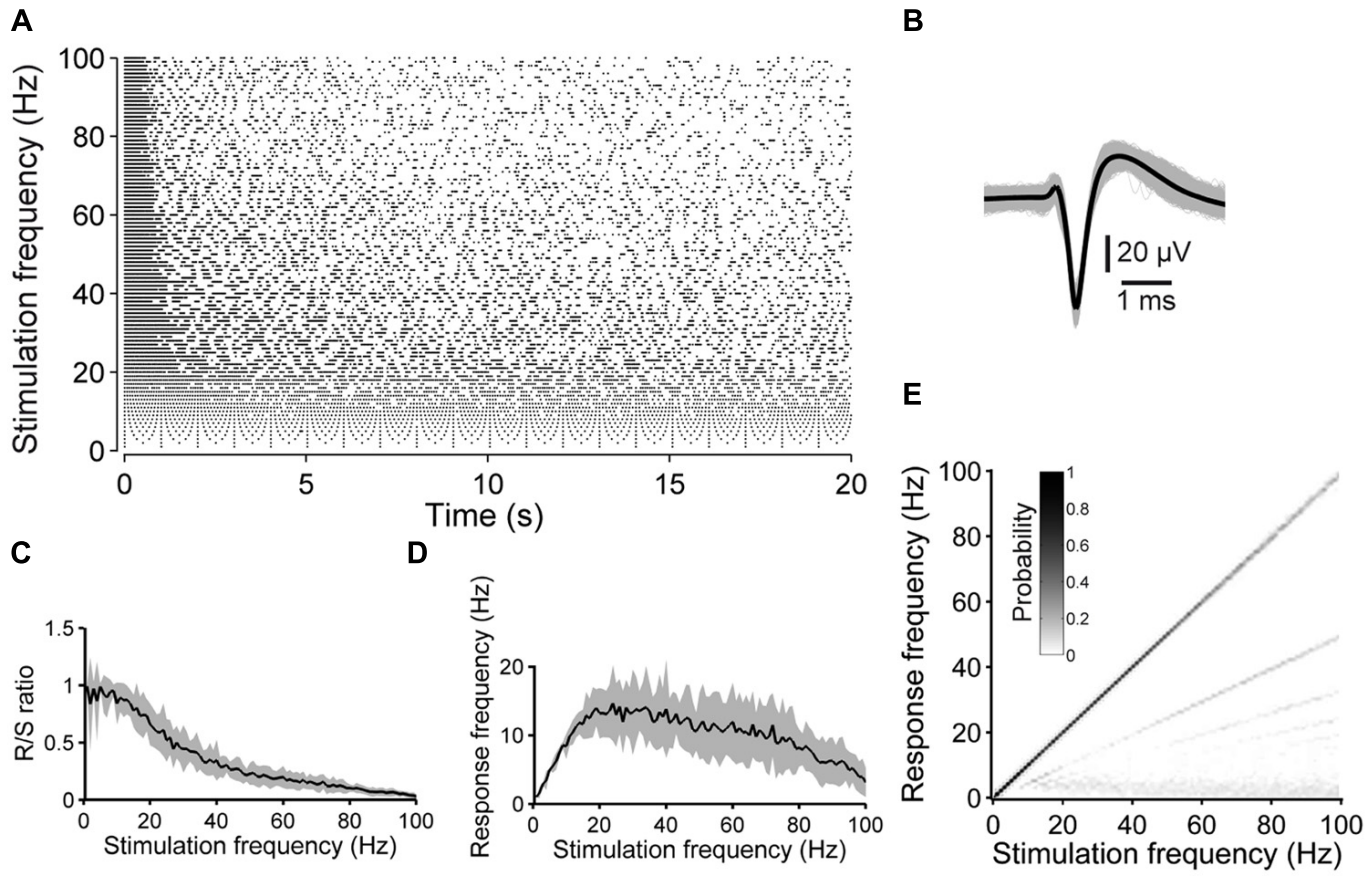
**Synaptically independent spike responses to 1–100 Hz stimulation. (A)** The raster plot shows an example of the spike responses to 20 s long pulse trains applied at various stimulation frequencies. **(B)** All ≈20000 spike waveforms detected during the experiment in **(A)** (gray: single spike, black: average). **(C)** R/S ratios and **(D)** mean spike frequency during the 20 s long stimulation periods as a function of the stimulation frequency, averaged over five experiments with four cultures (shaded areas indicate SEM). **(E)** The graph shows an estimation of the evoked frequency spectrum below 100 Hz of all stimulation blocks of the experiment shown in **(A)**. At high stimulation rates the neuron is not entrained by the stimulation pulses in a 1:1 manner anymore and, consequently, the main diagonal weakens and lines below the main diagonal become evident.

Frequency response graphs (F–R graphs; **Figure 1E**) were computed as follows. For each of the one hundred 20 s long stimulation blocks the ISIs were determined and converted to their corresponding frequencies in units of hertz. Histograms were computed (bin size 1 Hz) and, hence, peaks in the histograms give an estimate about the dominating frequencies, specifically, the relative probability of ISIs that correspond to a given spike frequency during a 20 s long stimulation block. The resulting histograms were plotted as gray scale graphs and vertically aligned at its corresponding stimulation frequency. ISIs smaller than 10 ms (i.e., frequencies above 100 Hz) were rarely observed with no systematic relationship to the stimulation frequency (not shown). The ordinate in **Figure 1E**, therefore, was truncated at 100 Hz.

#### Network Response to Pulses of Different Amplitudes

To test the efficacy of stimulus strength, to investigate the network excitability during the development and to obtain pulse amplitudes which can putatively lead to saturating responses, 20 current pulses of different amplitudes (μA): 5, 9, 13, 17, 21, 25 were applied to cultures at DIV: 7, 9, 12, 14, 16, 20, 22. Stimulation frequency was 0.1 Hz. The order of pulse amplitudes was randomized to avoid putative adaptation effects and to minimize the impact of slow spontaneous changes in the network excitability (see Results).

The amount of evoked activity is dependent on the site of stimulation, since the position of a given stimulating electrode relative to axons or cell bodies determines to which extend the electrical pulse can entrain the network. The choice of the stimulating electrode, therefore, was based on the SNR and number of spontaneous spikes. In all cultures, the electrode with the best SNR and maximum number of spontaneous spikes at DIV 7 was chosen as stimulating electrode throughout the development. In rare cases, the stimulating electrode was changed if an initially silent electrode with significantly better SNR and higher spontaneous spike rate appeared later during the development. This approach assures a functioning interface between the stimulating electrode and the network.

To investigate the impact of chronically absent fast GABAergic synaptic transmission on the electrically evoked activity, these experiments were performed with control cultures and age-matched sister cultures with chronically blocked GABA_A_ receptors.

To obtain **Figure 3**, the number of evoked spikes detected through all recording electrodes during the first 1000 ms post stimulus were trial-averaged and pooled separately for cultures with intact and with blocked fast GABAergic synaptic transmission for each age, culture and pulse amplitude.

#### Stimulation Frequency Dependence of Evoked Responses

To investigate the frequency dependence of the network response during different stages of network development, trains of 50 pulses with different frequencies were applied at DIV: 8, 13, 15, 19, 21. Pulse trains of different frequencies were in randomized order to avoid putative adaptation effects and the impact of slow changes in the excitability (see Results). The trains were intermittent by at least 60 s long periods of spontaneous network activity (i.e., considerably longer than recovery periods). To access the putative frequency dependence of the early responses, inter-pulse intervals (Δ*t*) were set to (ms): 50, 100, 150, 500, 1000, 5000, 15000 and the evoked activity during the first 20 ms post stimulus was analyzed.

Due to the bursting nature of the network activity, stimulation pulses can fall inside a reverberating burst response evoked by a previous pulse. In particular, in ≈3 week *in vitro* old cultures with blocked GABA_A_ receptors, the fraction of pulses that fall into reverberating bursts can be relatively high at short Δ*t*. Therefore, trials were neglected in the analysis if spikes detected 30 ms pre stimulus originated from more than three active electrodes, indicative for a population burst. In this context, an electrode was considered as active if two or more spikes were detected 30 ms pre stimulus. In cultures with blocked GABA_A_ receptors, the fraction of trials considered can be as low as ≈10–30% at Δ*t* = 50 ms. On average, 64 and 97% of the trials were considered in the analysis for Δ*t* = 50–150 ms and Δ*t* = 500–15000 ms, respectively. In controls, on average 75% (minimum 35%) and 97% of the trials were considered in the analysis for Δ*t* = 50–150 ms and Δ*t* = 500–15000 ms, respectively. To analyze the late reverberating burst responses evoked by individual pulses, Δ*t* of 1, 5, and 15 s were considered only, because the evoked spike bursts can outlast 100s of milliseconds. Single pulses were considered to evoke a reverberating burst response if at least 30 spikes originating from >3 electrodes occurred 21–1000 ms post stimulus and if spikes detected 30 ms pre stimulus originated from ≤3 electrodes.

#### Double-Pulse Experiments

To investigate the time range during which consecutive pulses interact and to study network refractoriness, two consecutive stimulation pulses (conditioning and T-Pulse; C- and T-pulse, respectively), separated by various time intervals (ms): 1, 2, 4, 6, 8, 10, 20, 40, 60, 80, 100, 200, 400, 600, 800, 1000, 5000, 15000 were applied. Each pulse pair was applied 30 times in pseudo-randomized order. Preliminary studies suggested that there is little interaction of consecutive pulses if 10 s or more separate them. Therefore, after each T-pulse there was a 15000 ms period without stimulation before the next randomly chosen C–T pulse pair was applied. After subtracting the average response elicited by single pulses, the amount of evoked activity by the T-pulse during the first 200 ms post stimulus was analyzed (**Figure 6A**). During this time window, the network response of cultures with intact GABAergic transmission and the first wave of activity in cultures with blocked fast GABAergic synaptic transmission was largely decayed (see Results).

#### Prolonged Low-Frequency Stimulation

To investigate response properties during ongoing changes of the overall network activity, cultures were probed for 1 h with electrical pulses at 1 Hz.

#### Statistics

The choice of the statistical test depended on whether the data were normally distributed or not. Normality was assessed using Lilliefors goodness-of-fit test. A data set that produced a significant result at alpha = 0.05 was considered to be non-normal. Statistical tests of the difference between a group mean and 0 were performed with Student’s *t*-test for normal data and Wilcoxon signed-rank test for non-normal data. Statistical tests of differences between two groups of normal data were performed using Student’s *t*-test. Tests between two groups of non-normal data were performed using the Mann–Whitney *U* test. If not stated otherwise, a group represents a set of either control cultures or a set of cultures, where GABA_A_-receptors were chronically blocked. Data are presented as means ± SEM.

## Results

The input–output relationships of cultured cortical networks were investigated during the first 3 weeks of their *in vitro* development. We focused on the alterations of these input–output relationships during the maturation as a result of impaired GABA_A_ergic synaptic transmission. Furthermore, several protocols were applied to characterize the general electrically evoked network response of ‘mature’ networks (i.e., cultures older than 3 weeks *in vitro*).

### Spontaneous Activity and General Culture Properties

All cultures with intact GABAergic synaptic transmission underwent a typical development of spontaneous activity with regular population bursting, starting at about the end of the first week *in vitro* (for developmental course see for example Figures 1A and 2A in Baltz et al. (2010) and also Wagenaar et al. (2006a)). Network activity, then, became more heterogeneous with periods of higher and lower burst activity after about 2 weeks *in vitro* (see also Figure 7A below, for an example of a “mature” activity pattern). On the contrary, cultures with GABA_A_R blockade retain a stereotyped and much synchronized bursting pattern (see Figures 7A and 2B in Baltz et al. (2010) and also below). In both network types, GABAergic cells with a large soma area and long, purely ramified dendrites dominate the GABAergic population [see Figure 6A in Baltz et al. (2010)]. These parvalbumin expressing cells innervate their postsynaptic cells in a basket-like fashion *in vitro*, are born early during the embryonic development of the rat (around embryonic day 13) and reside in the subplate at birth (Voigt et al., 2001).

### Direct Responses

First, the range of frequencies during which extracellular stimulation can reliably evoke direct neuronal responses under the present experimental conditions was investigated. To this end, neurons were pharmacologically isolated in cultures aged between 23 and 36 DIV by applying the synaptic blockers CNQX (50 μM), D-AP5 (50 μM) and bicuculline (10 μM) to block AMPA, NMDA, and GABA_A_Rs, respectively. Twenty-second long pulse trains were applied with pulse frequencies ranging from 1 to 100 Hz through a single electrode in pseudo-random order (see Materials and Methods).

The ratio of responses to the number of stimulus pulses (R/S ratio) and the spike latency were strongly frequency-dependent. At low stimulation rates (≈1–10 Hz), the R/S ratio was near unity (**Figures 1A,C**). At stimulation rates significantly higher than ≈10 Hz, however, evoked responses became increasingly unreliable during the pulse trains, and the R/S ratio systematically decreased to, on average, 3.11 ± 0.87% at 100 Hz (*n* = 5 experiments with 4 cultures) (**Figure 1C**).

**FIGURE 2:**
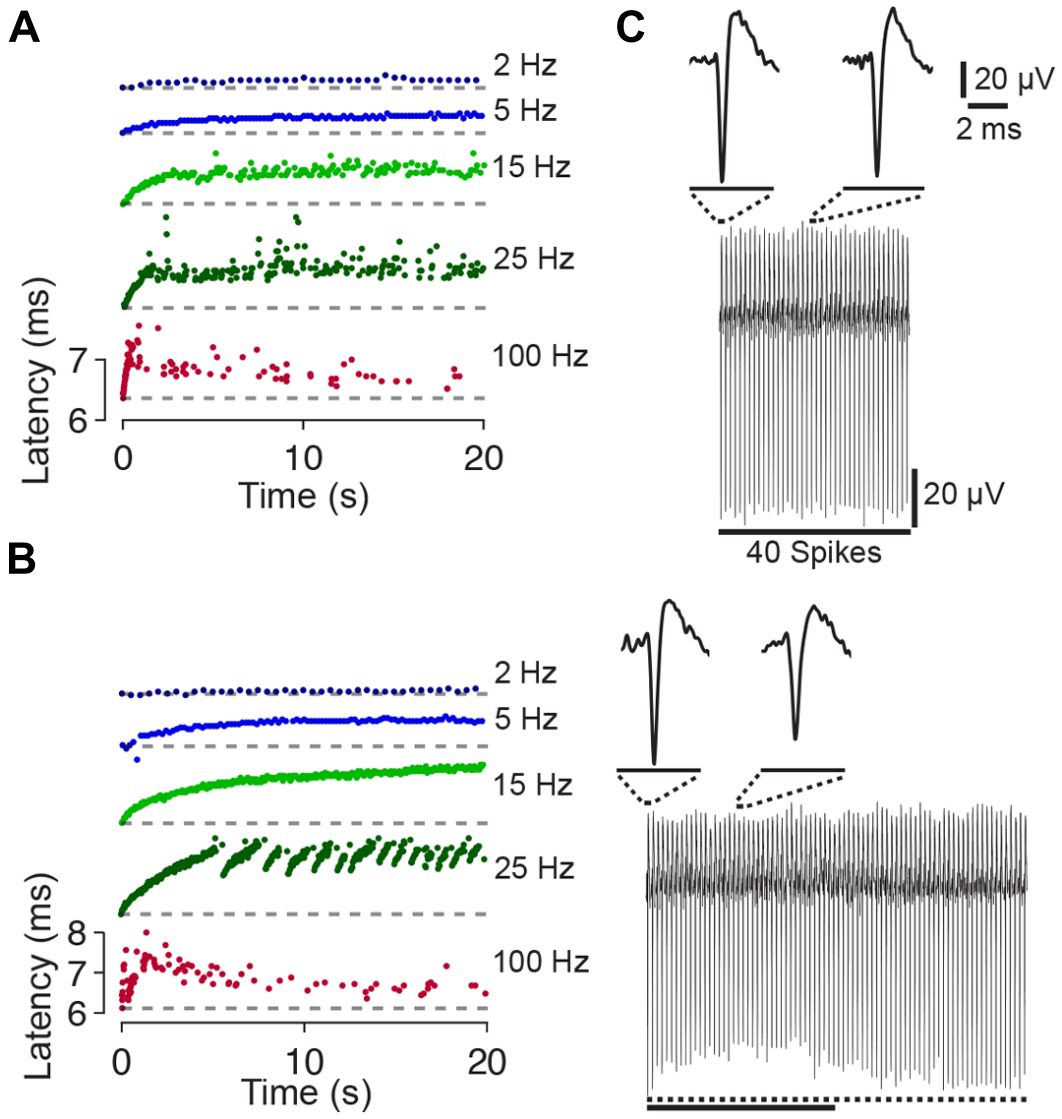
**Latency and amplitude change of synaptically independent spike responses to 1–100 Hz stimulation. (A)** Spike latency as a function of time during 20 s long stimulation blocks. The raster plots for the experiment shown in **Figure 1A** indicate a change of the spike latency during different periods of stimulation. Colors code stimulation frequency (see labeling on the right). The change of the spike latency depends on the stimulation frequency. Note that each raster plot was shifted in *y*-direction for clarity (i.e., first spike, at time index zero, always occurs with a relatively short latency post stimulus; ≈6.3 ms; the latency of the first spike is shown as dashed line). **(B)** Another experiment with abrupt changes of the spike latency at the stimulation frequency of 25 Hz. **(C)** (Top) All 40 spike wave forms of the experiment shown in **Figure 1A** during 2 Hz stimulation are stacked from left (spike evoked by the first pulse) to the right (spike evoked by the last pulse). Insets show an enlarged view of the first and twentieth spike. No major differences in spike shape or amplitude become apparent. (Bottom) Same as above but during a 100 Hz stimulation block. During ongoing stimulation, the spike amplitude decreases and then recovers. Dotted line below the stacked spikes indicates the amplitude of the first spike. The solid line indicates 40 spikes (same scale as above).

During the first 10–20 pulses of a train the spike latency typically increased (up to ≈1–2 ms). Considering the increase in latency, two response types could be distinguished. The first response type was observed in three out of five cases and was characterized by a steady latency increase (**Figure 2A**). With ongoing stimulation, the spike latency either remained relatively stable (at stimulation rates <<100 Hz), or spikes were evoked rarely with varying latency when pulse rates approached 100 Hz. In the remaining two cases, the second response type was characterized by latency increases which abruptly recovered and increased again (**Figure 2B**). Recently, it was hypothesized that the different behavior in response to extracellular stimulation refers to distinct neuronal subtypes [e.g., fast spiking or bursting, Gal et al. (2010)].

The amplitudes of the initial 20 spikes and the spike latency were slightly negatively correlated (**Figure 2C**), with a correlation coefficient significantly smaller than zero (–0.29 ± 0.11; *p* < 0.05). Smaller spike amplitudes might indicate an incomplete recovery of the sodium conductance from previous spiking (see Discussion).

To estimate at which frequencies the neurons spike during the 20 s long stimulation blocks, time-independent frequency–response graphs (F–R graphs) were determined from the ISI distributions (see Materials and Methods). In case a neuron responds in a 1:1 manner to each electrical pulse, independent from the stimulation frequency, a black main diagonal would be present in F–R graphs only. On the other hand, subthreshold membrane oscillations could lead to several spikes in responses to a single stimulation pulse, when a neuron is excited at its preferred frequency. The latter could be indicative above the main diagonal in F–R graphs. Both cases were, however, not observed under the present experimental conditions. In contrast, F–R graphs revealed the tendency of neurons not to respond to every single pulse at higher stimulation frequencies, but to every second, third and so forth, which became evident by the second, third and so forth lines below the main diagonal, respectively (**Figure 1E**).

The average spike frequency during stimulation blocks was determined and plotted as a function of the stimulation frequency. The average spike frequency of the 20 s long pulse trains did not exceeded 20 Hz, and reached its maximum at a stimulation frequency of ≈20 Hz (**Figure 1D**).

In addition to earlier reports (Jimbo et al., 2000; Wagenaar et al., 2004; Gal et al., 2010), these data show that the reliability and the latency of direct responses systematically depend on the frequency and duration of applied pulse trains at frequencies in the range between 1 and 100 Hz.

### Dependency of Electrically Evoked Responses on Pulse Amplitudes during the Development

Response properties of cortical networks depend on many parameters, such as synaptic coupling strength, connectivity and the differentiation of neuronal cell types, to name only a few. These parameters considerably change during network maturation. To determine changes in network excitability during the development, cultures were probed with pulses of various amplitudes (5–25 μA at 0.1 Hz) at different DIVs. The inhibitory action of fast GABAergic synaptic transmission is expected to develop gradually during the third week in culture [see Baltz et al. (2010)]. To assess the contribution of fast GABAergic synaptic transmission on the electrically evoked activity during the development, similar experiments were performed with age-matched sister cultures, in which GABA_A_Rs were chronically blocked by 20 μM gabazine, added shortly after culture preparation.

**FIGURE 3:**
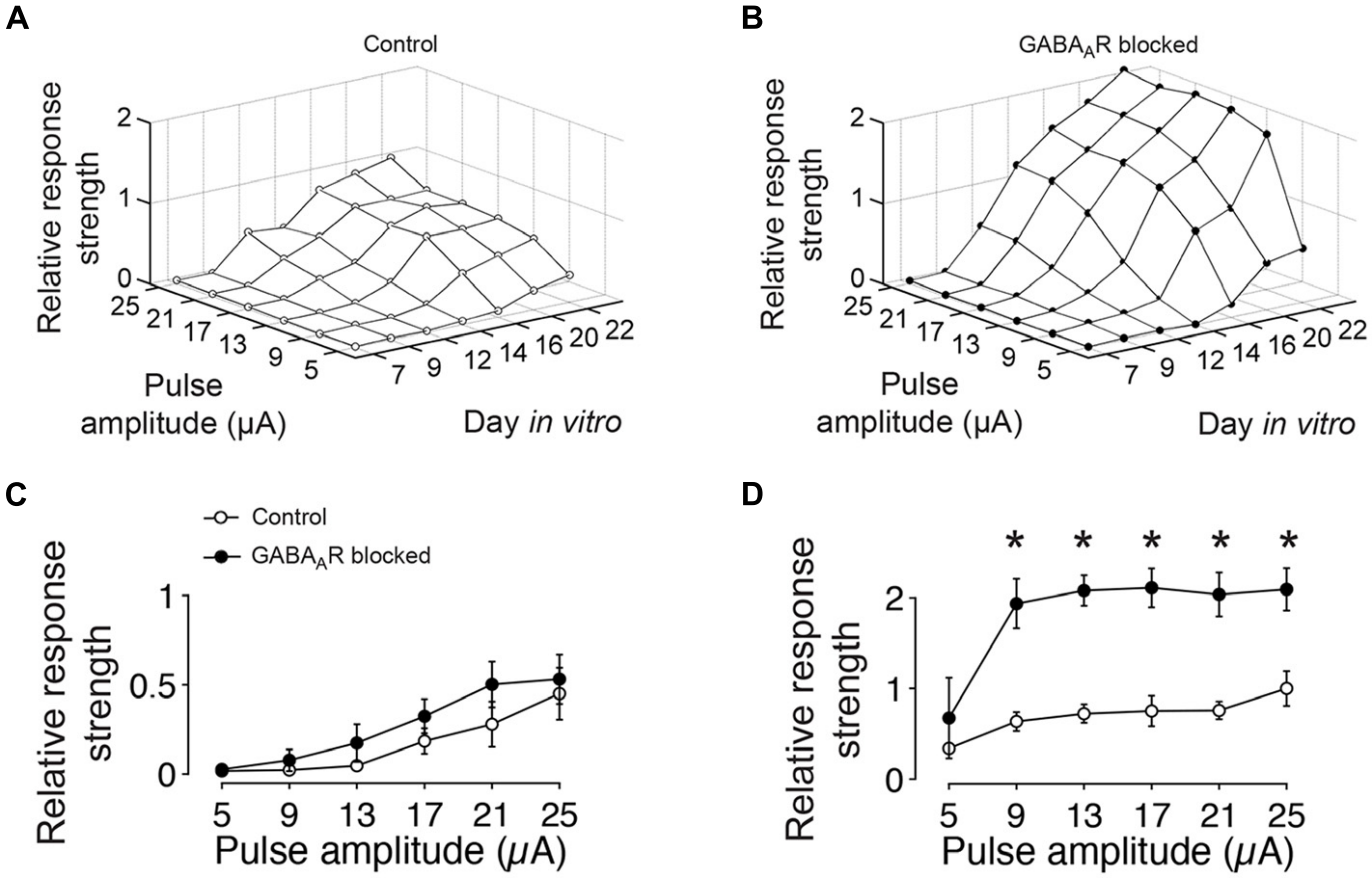
**GABA-dependent differences in the amount of evoked spikes to extracellular current pulses of different amplitudes. (A)** The graph shows the relative amount of evoked spikes to low-frequency (0.1 Hz) electrical stimulation with different pulse amplitudes and at different DIV (average of four cultures). The graph is normalized to its maximum at DIV 22 and 25 μA. **(B)** Same as **(A)** but for age-matched sister cultures with chronically blocked GABA_A_ergic transmission (*n* = 4). The graph is normalized to the maximum of controls (DIV 22 and 25 μA in **A**). **(C)** Differences between cultures with (filled circles) and without blocked (empty circles) GABA_A_ergic synaptic transmission were not significant at DIV 12. **(D)** After 22 DIV, differences were significant (*n* = 4 cultures each group; asterisks indicate significance; *p < 0.05).

At the earliest age studied (DIV 7), extracellular electrical stimulation evoked very few spikes, irrespective of the stimulation amplitude or the presence or absence of GABAergic synaptic transmission. With ongoing development, the amount of evoked activity increased for both conditions and was highest in about 3 week old cultures with blocked GABA_A_Rs (**Figure 3**). After 12 DIV, the amount of evoked spikes in blocked and unblocked cultures did not differ significantly (**Figure 3C**). In older cultures, however, significantly more spikes were evoked for tested stimulation amplitudes greater than 5 μA in cultures with blocked GABA_A_Rs (**Figure 3D**). At 22 DIV, the maximum tested pulse amplitude evoked 109.15 ± 23.31% more spikes in gabazine-treated cultures compared with age-matched controls (*n* = 4 cultures each group) (**Figure 3D**).

In summary, these results show a continuous increase of the evoked activity during the first 3 weeks of development for both, cultures with and without functional fast GABAergic synaptic transmission. Differences in the total amount of evoked spikes between cultures with intact and blocked fast GABAergic synaptic transmission became apparent at the end of the second week *in vitro*, when the amount of evoked activity in GABA_A_Rs blocked cultures exceeded that of unblocked cultures.

### Frequency Dependent Responses during Development

During the experiments discussed in the previous section, the cultures were stimulated with different pulse amplitudes, but at a fixed low frequency. In this section, neuronal activity evoked by pulse trains of a fixed amplitude (25 μA) is considered, but applied at different frequencies. The cultures were stimulated at different time points during the development and, similar to the experiments above, the experiments were performed in control cultures as well as with age-matched sister cultures, where GABA_A_Rs were chronically blocked.

**FIGURE 4:**
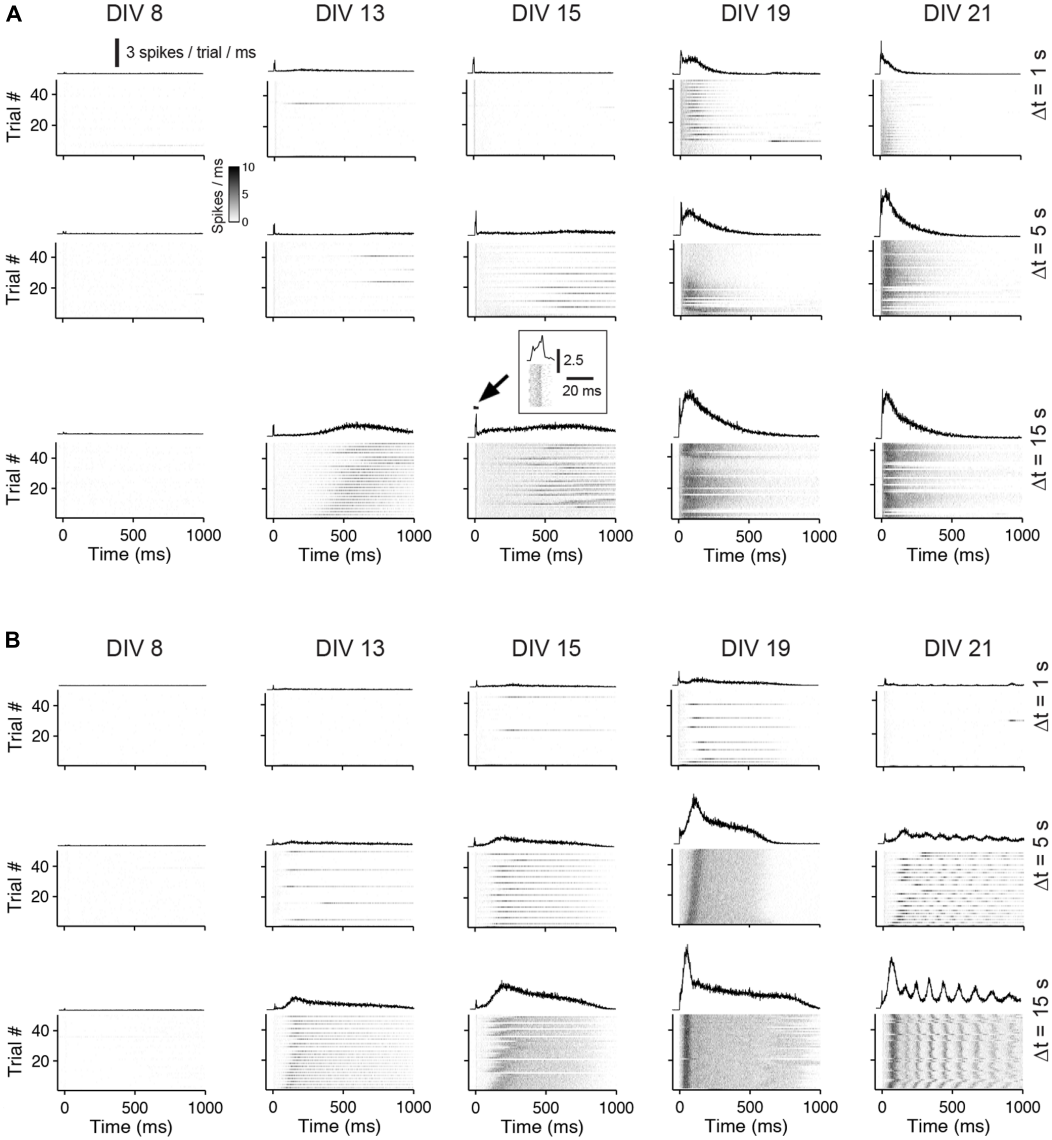
**Network responses to pulse trains of different frequencies during the development. (A)** The gray scale graphs show the network responses to individual pulses applied at different inter-pulse intervals (Δ*t* = 1, 5, or 15 seconds) and DIV (top labeling) for the same culture (bin size is 1 ms). The line graph above each gray scale graph shows the trial-averaged responses. Trials that fall in the burst responses of a previous pulse were omitted. The inset shows the early response at higher time resolution. **(B)** Same as **(A)** except for a culture with GABA_A_ergic transmission being chronically blocked.

Generally, the number of evoked spikes increased with development for all stimulation frequencies and was highest in about 3 week old cultures. On average, the highest number of spikes was evoked at lowest stimulation rates (i.e., inter-pulse intervals of 15 s) in 19 DIV old cultures with blocked GABA_A_Rs. In these cultures, on average, 180.43 ± 32.13% more spikes were evoked compared with unblocked age-matched controls (*n* = 4 cultures each group). The increase of the overall evoked activity with development was mainly a result of the emerging late network-wide burst response during the development (**Figure 4**).

In cultures older than 2 weeks, the temporal evolution of the evoked activity differed markedly between cultures with and without GABA_A_R blockade. In control cultures, the burst response typically decayed after an initial rising phase. The burst response of cultures with GABA_A_R blockade, in contrast, often had multiple rising and decay phases (compare **Figure 4A** with **Figure 4B** at 21 DIV and Δ*t* = 15 s).

The early response to electrical stimulation (up to 20 ms post stimulus) mainly reflects direct neuronal excitation (see above) and early postsynaptic spikes (Jimbo et al., 2000; Marom and Shahaf, 2002; Wagenaar et al., 2004), and the late response (21–1000 ms) is dominated by synaptically mediated reverberating network-wide bursts. Both aspects of the stimulus response are considered separately in the next paragraphs.

The amount of spikes during the early phase generally increased with development for all pulse frequencies. No statistically significant differences were found in the amount of evoked spikes during the early phase between cultures with and without GABA_A_R blockade (not shown).

**FIGURE 5:**
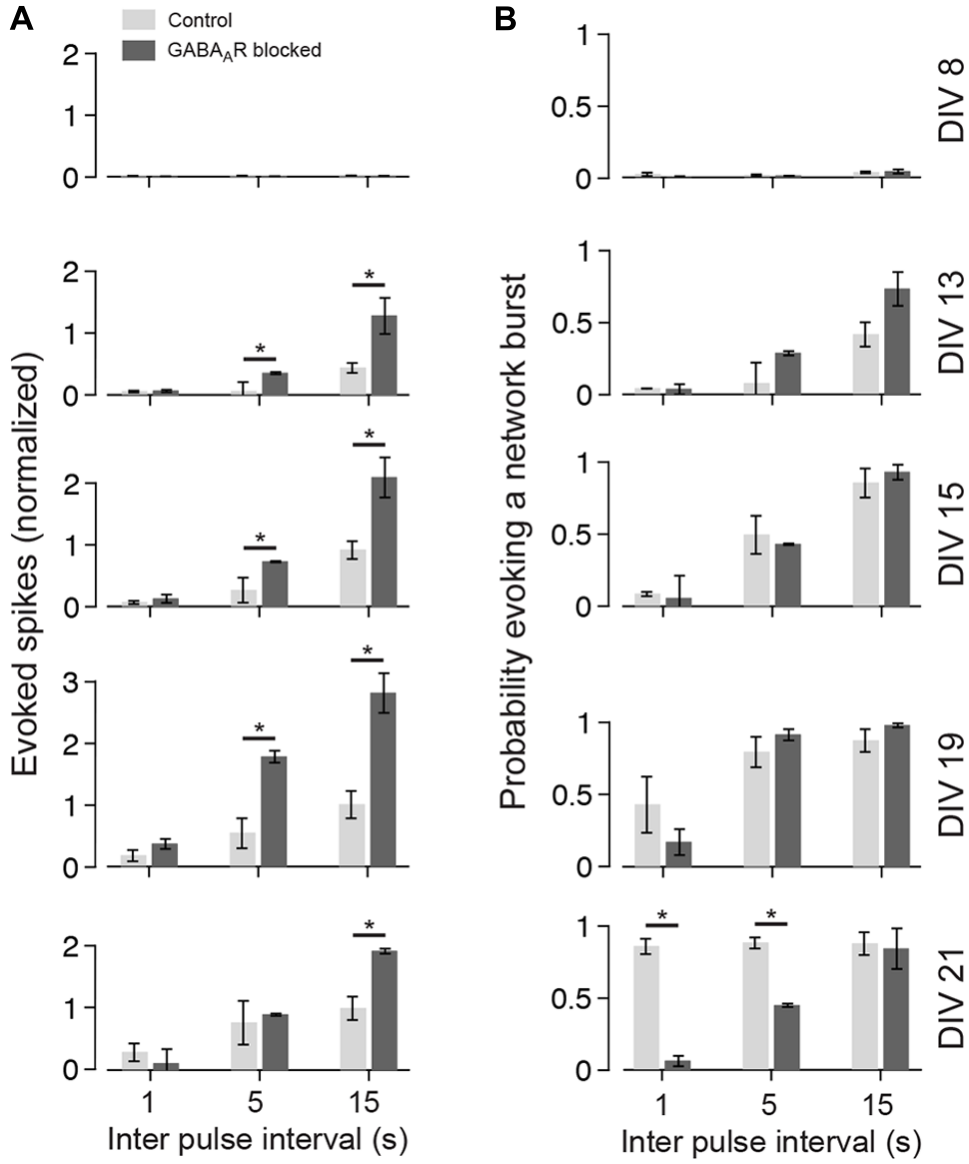
**Summary graph for the development of the late responses. (A)** The bar plots show the amount of evoked spikes during 21–1000 ms post stimulus in response to various pulse frequencies (light gray controls; dark gray chronically blocked GABA_A_Rs) at different DIV (see labeling on the right in **B**). The graphs are normalized to the maximum of control cultures (DIV 21; Δ*t* = 15000 ms). **(B)** Same as **(A)**, but for the probability of evoking a network burst (*n* = 4 each group; asterisks indicate significance; *p < 0.05).

To analyze the late response for individual pulses separately, inter-pulse intervals greater than 500 ms were considered (i.e., Δ*t* = 1, 5, and 15 s). The interaction of two consecutive pulses for lower Δ*t* is discussed separately below. Generally, lowest stimulation frequencies (Δ*t* = 5 and 15 s) regularly evoked strong network bursts in an all-or-none manner in cultures with blocked GABA_A_Rs aged about 3 weeks *in vitro* (e.g., **Figure 4** at DIV 21 and Δ*t* = 5 s), which typically contained more spikes than an evoked network burst in age-matched cultures with intact GABAergic transmission (**Figures 4** and **5A**). After about 3 weeks *in vitro*, bursts were rarely evoked when Δ*t* was reduced to 1 s in cultures with blocked GABA_A_Rs (**Figures 4B and 5B**). In contrast, bursts were evoked with high reliability in age-matched cultures with intact GABA_A_ergic synaptic transmission (**Figure 4A** at 21 DIV and Δ*t* = 1 s, **Figure 5B**). These burst, however, contained fewer spikes, compared with the all-or-none bursts of blocked cultures. These differences between cultures with and without intact fast GABAergic transmission already became indicative in individual cultures at DIV 19 and became statistically significant after 21 DIV (**Figures 4** and **5B**).

In some cases, a systematic latency increase of the reverberating burst response was observed during ongoing stimulation (e.g., **Figure 4B** at 19 DIV and Δ*t* = 5 s), which might indicate an incomplete recovery from activity-dependent resources (e.g., from synaptic depression; see Discussion).

Taken together, strongest responses were evoked in about 3 week old cortical cultures with low-frequency stimulation in all cultures. The susceptibility to low-frequency stimulation is indicative of a network-wide refractory period as a result of sustained bursts firing. Furthermore, fast GABAergic synaptic transmission, if mature, enables the network to respond to stimuli of higher rates, but with a reduced number of spikes compared to the responses of cultures with blocked GABA_A_Rs. This issue is investigated further in the next section.

### Double-Pulse Experiments

To investigate the time range during which consecutive stimuli interact, ‘mature’ (i.e., 21–26 DIV old) cortical cultures were probed with two pulses, separated by various time intervals (Δ*t* = 1 ms to 15 s; see also Materials and Methods). As before, these experiments were performed in cultures with and without intact fast GABAergic synaptic transmission.

**FIGURE 6:**
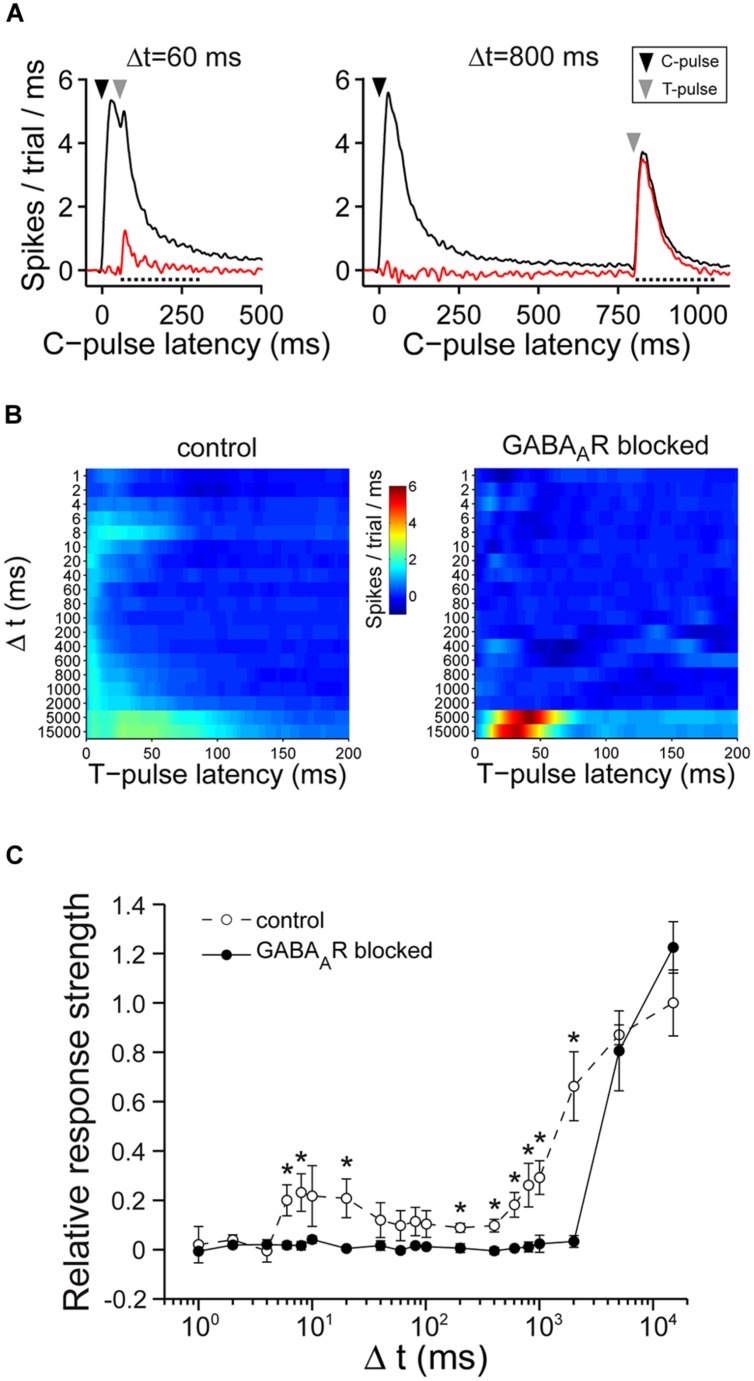
**Double-pulse experiments. (A)** (Left) Shows an example of the average responses before (black) and after (red) the subtraction of the average response to single pulses for Δ*t* = 60 ms. The dotted line indicates the time window of the first wave of activity post stimulus (200 ms) which was considered for the analysis. (Right) Similar graph to the one on the left except for Δ*t* = 800 ms. **(B)** The graph shows the spikes post stimulus the T-pulse, after subtraction of spikes that were evoked by single pulses for one culture without (left) and with (right) chronically blocked GABA_A_Rs. **(C)** The summary graph shows the T-pulse responses. The graph is normalized to the average responses of cultures with intact GABA_A_ergic synaptic transmission at Δ*t* = 15000 ms (*n* = 7 control cultures; *n* = 6 cultures with chronically blocked GABA_A_ergic synaptic transmission; 21–26 DIV; asterisks indicate significant differences between control and blocked cultures; *p < 0.05).

The first pulse (conditioning, C-pulse) typically evoked a strong reverberating synaptic network-wide burst response. The response to the second pulse (test, T-pulse), then, varied systematically with the inter-pulse interval. To estimate the amount of activity evoked by the T-pulse, the average response evoked by single pulses (i.e., pulses with Δ*t* = 15 s) was subtracted, and the amount of evoked activity shortly after the T-pulse was analyzed (**Figure 6A**).

Cultures with intact GABAergic synaptic transmission showed a broad spectrum of responses evoked by the T-pulse. Generally, the T-pulse evoked more spikes for various Δ*t* compared to cultures where fast GABAergic transmission was chronically blocked (**Figures 6B,C**). Particularly, for Δ*t* around 10 ms and Δ*t* = 200–2000 ms, the spike number evoked by the T-pulse was significantly higher compared to blocked cultures (**Figure 6C**). Moreover, excitability gradually recovered on a relatively low time scale, i.e., for Δ*t* greater than 10–100 ms in cultures with intact GABA_A_ergic transmission (**Figures 6B,C**). In contrast, in cultures with blocked GABA_A_ergic synaptic transmission, there was a prolonged refractory period of at least 2 s (**Figures 6B,C**) during which the T-pulse was almost ineffective. The evoked activity, then, recovered for Δ*t* ≥ 5 s (**Figures 6B,C**).

These data indicate that cultures with and without functional GABAergic transmission show prolonged periods of low excitability after previous synaptic activity, but with different, GABA-dependent, time ranges of recovery.

### Slow Changes of the Network Excitability

Previous experiments have shown that in about 3 weeks *in vitro* old cortical cultures, network dynamics can comprise recurring minute long periods of lower and higher activity (Wagenaar et al., 2006a,b; Baltz et al., 2010) which occur spontaneously and are abolished when GABAergic transmission is blocked (Baltz et al., 2010).

**FIGURE 7:**
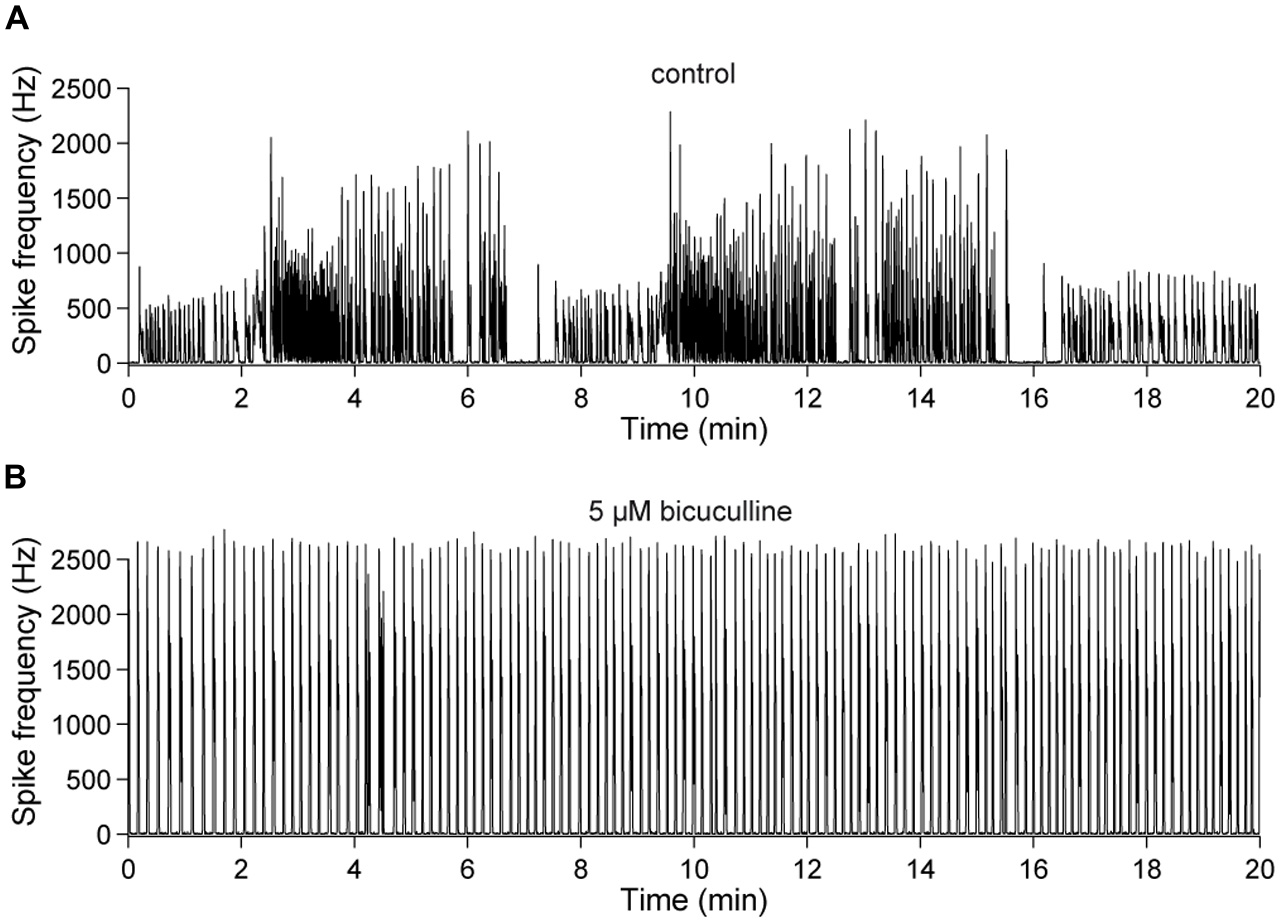
**Slow changes of the spontaneous network activity are mediated by GABA_A_ergic synaptic transmission. (A)** Spontaneous population activity of a 22 DIV old cortical culture under control conditions. The line-graph indicates the global firing rate, which is defined as the number of detected spikes through all electrodes per time unit (second). The slow change of the overall network activity was abolished by an **(B)** acute blockade of GABA_A_Rs (bicuculline, 5 μM).

This observation was quantified in 20 min long recordings of the spontaneous network activity of 23–28 DIV old cultures (**Figure 7**). Periods of low activity occurred, on average, every 4.06 ± 0.27 min (≈0.004 Hz) under control conditions and were abolished in all cultures in the presence of bicuculline (*n* = 5) (**Figure 7B**).

To investigate the excitability during such spontaneous changes of the network activity, cultures aged older than 3 weeks were probed with electrical pulses applied at 1 Hz for one hour (i.e., 3600 pulses in total). Again, similar experiments were performed with cultures with and without functional GABA_A_R-mediated synaptic transmission.

**FIGURE 8:**
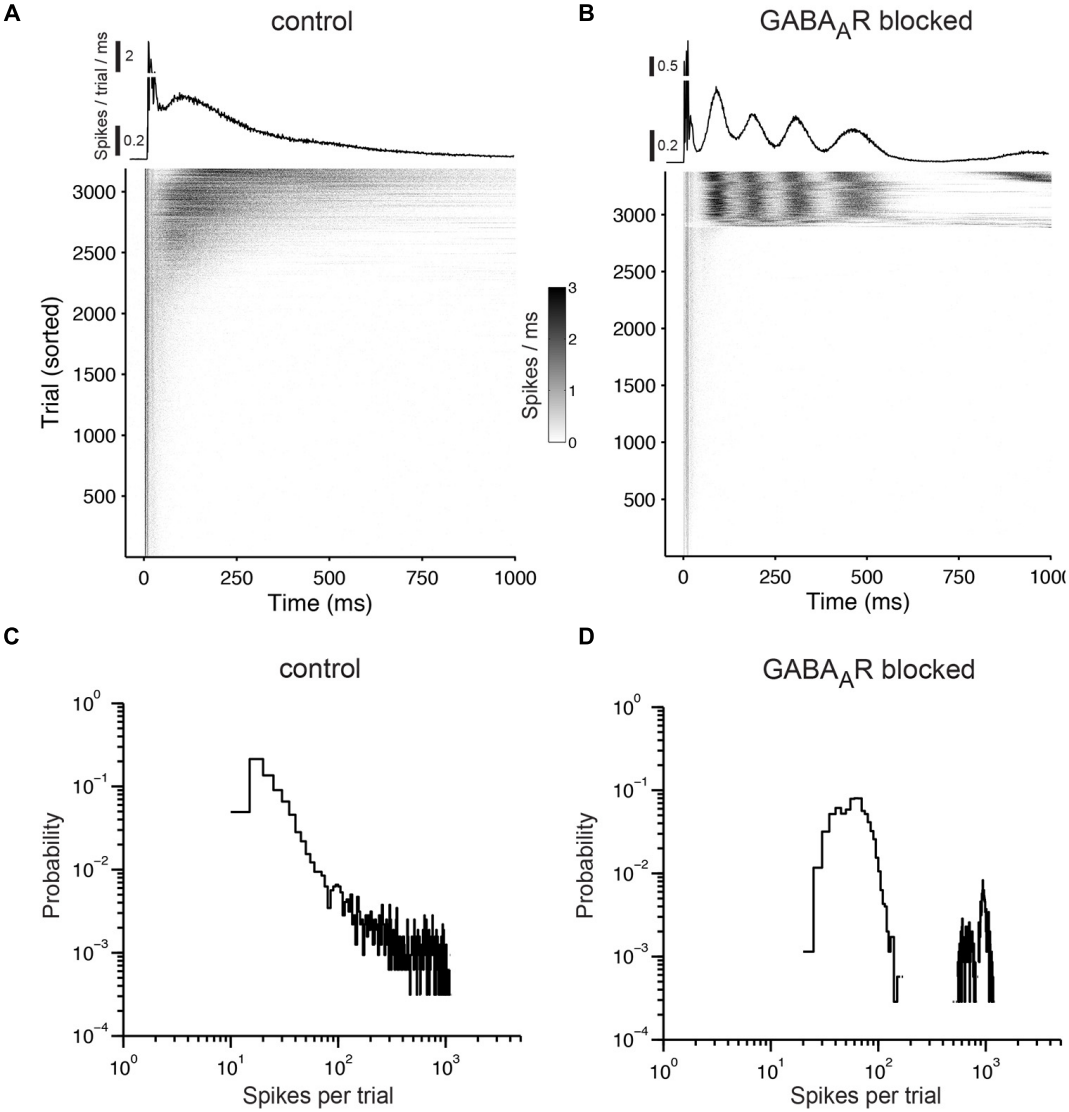
**Responses to prolonged electrical stimulation. (A)** The top graph shows the trial average of the population responses to electrical stimulation at 1 Hz. The early responses are cut off at 0.5. The gray scale graph below shows the population responses for each stimulation pulse (trials that fall within a network burst were not considered and were omitted). Each gray dot reflects the number of evoked spikes for a 1-ms wide bin. The graph is sorted by the number of evoked spikes. Note that virtually all activity in the network is time-locked to the stimulation pulses. **(B)** Same as **(A)** except for a culture with chronically blocked GABA_A_Rs. **(C)** Distribution of the number of evoked spikes during prolonged electrical stimulation. Cultures were stimulated for one hour at 1 Hz under control conditions and **(D)** with chronically blocked GABA_A_Rs. Similar results were obtained in six cultures per group aged between 22 and 36 DIV (see main text for details). The bin width is five spikes.

**FIGURE 9:**
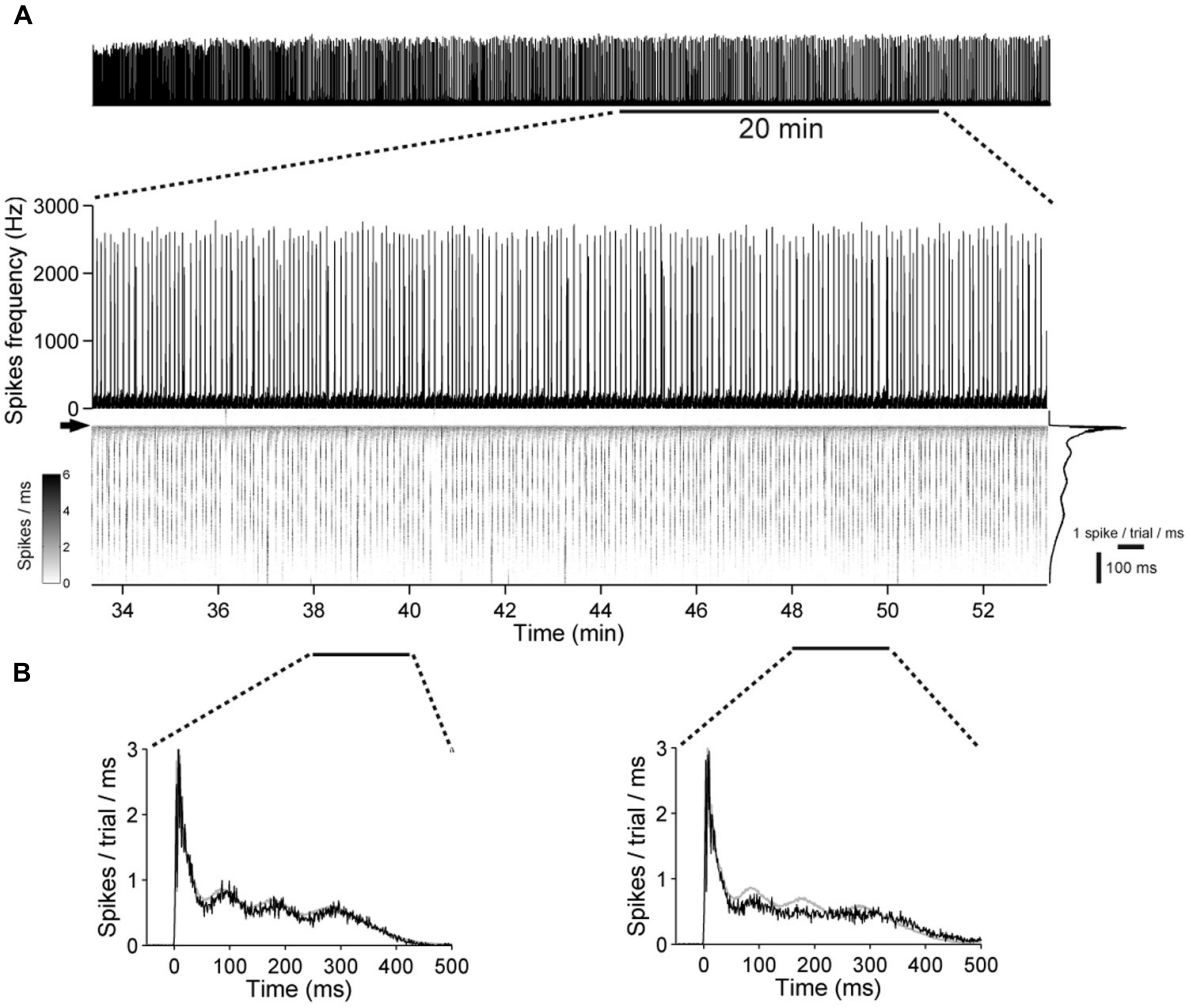
**Low variability in the network excitability during prolonged stimulation in networks with chronical absence of GABA_A_R mediated synaptic transmission. (A)** (Top) A 21 DIV old culture during a 1 h stimulation period, at 1 Hz. (Bottom) A 20-min long period is shown enlarged. The gray scale graph shows the evoked responses ranging from 50 ms before up to 500 ms after each stimulation pulse, temporally aligned to the line graph above. The arrow denotes the time point of stimulation. Trial-averaged population response is shown on the right. **(B)** Trial-averaged population response of the network during 2 min long time intervals (black: response during the indicated interval; gray: all trials).

**FIGURE 10:**
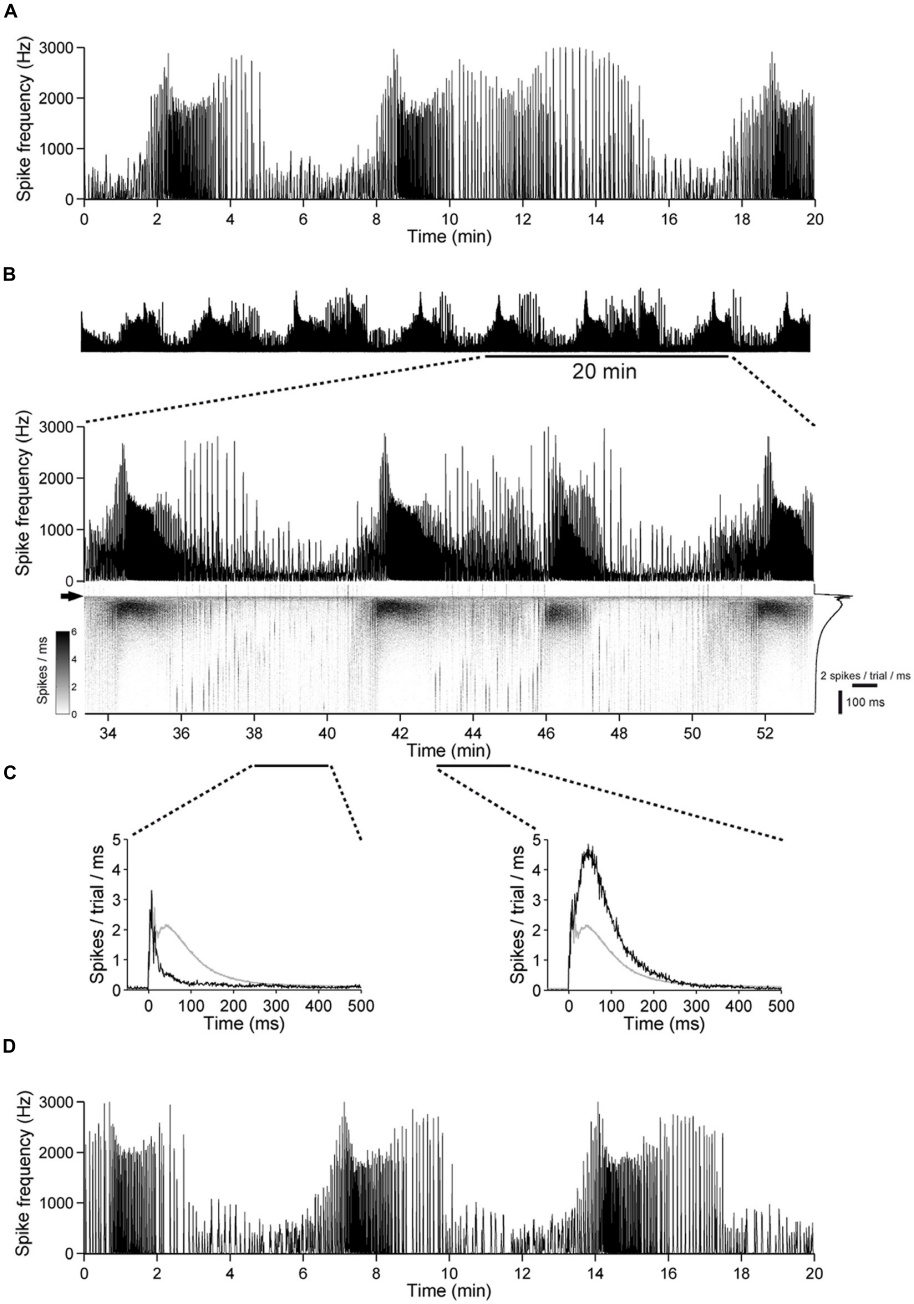
**The excitability undergoes slow changes in networks with intact GABAergic synaptic transmission. (A)** Spontaneous population activity of a 22 DIV old cortical culture. The amount of spike activity undergoes recurrent, spontaneous slow changes over time. **(B)** (Top) The same culture as in **(A)** during a 1 h stimulation period, at 1 Hz. (Bottom) A 20-min long period is shown enlarged. The gray scale graph shows the evoked responses ranging from 50 ms before up to 500 ms after each stimulation pulse, temporally aligned to the line graph above. The arrow denotes the time point of stimulation. Trial-averaged population response is shown on the right. **(C)** Trial-averaged population response of the network during 2 min long time intervals (black: response during the indicated interval; gray: all trials). **(D)** Spontaneous activity of the culture in **(A–C)** after the stimulation experiment.

When stimulating cultures with chronically blocked GABA_A_Rs at 1 Hz, the pulses either elicited a few spikes shortly after each pulse or, infrequently, a strong reverberating synaptically mediated burst response (**Figure 8B**) leading to a bimodal distribution in the histograms of evoked spikes (**Figure 8D**) and a relatively constant network response (**Figure 9**). In contrast, cultures with intact GABAergic synaptic transmission showed a much broader spectrum of evoked activity (**Figure 8A**). The distribution of the number of elicited spikes was in some cases almost exponential (**Figure 8C**). However, the amount of evoked spikes strongly varied with the very slow oscillation of the overall network activity (**Figure 10**). The systematic variation of the number of evoked spikes indicates that periods of higher and lower spontaneous network activity reflect periods of higher and lower network excitability. These slow changes of the excitability, then, can provide “power-law-like” response characteristics.

The number of periods with higher and lower network activity was not altered markedly by electrical stimulation (**Figures 10A,D**). Moreover, most of the network activity was time-locked to the applied pulses (**Figures 8A and 10B**), indicating that the pulses predominantly act as a trigger for the otherwise spontaneously occurring spike bursts and that the slow change of the excitability is not affected strongly by electrical stimulation.

To investigate whether this is a result of chronic over-excitation or acute absence of GABA_A_R mediated synaptic transmission, we raised additional cultures for 3 weeks and performed a similar experiment before and after acute application of gabazine (100 μM). Similar to above results the slow variations of the excitability were abolished (*n* = 3, not shown), suggesting that slow variations of the network excitability are the result of the interplay between glutamatergic excitation and GABAergic inhibition.

It has been shown that sub-micromolar concentrations of gabazine selectively block phasic inhibition while leaving tonic GABA action unaffected (Semyanov et al., 2003; Farrant and Nusser, 2005). To estimate whether tonic or phasic inhibition dominates we performed again similar experiments before and after application of 0.5 μM gabazine to the culture medium (*n* = 2). Similar to all experiments in this set, slow variations of the excitability were abolished, indicative for a stronger role of phasic over tonic GABA action.

Taken together, most of the variations of responses to electrical stimulation of cortical networks *in vitro* occur spontaneously and seem to be a result of an underlying slow oscillation of the network excitability induced phasic GABA_A_R-mediated synaptic transmission and does not seem be induced by electrical stimulation itself.

## Discussion

To study the general output properties of cortical networks *in vitro* in response to short extracellularly applied electrical pulses, the networks were probed by various stimulation protocols at different time points during the first 3 weeks of their *in vitro* development. Special attention was paid to the impact of absent fast GABAergic neurotransmission during the network maturation. The total amount of electrically evoked activity increased during the first 3 weeks *in vitro* in cultures with intact and with chronically blocked fast GABAergic transmission. Under both conditions, the maximum output firing rate was reached only with the lowest stimulation frequency tested [i.e., inter-pulse intervals (Δ*t*) of 15 s].

In ≥3 weeks *in vitro* old control cultures, the amount of electrically evoked activity seems to be governed by at least two, possibly mutually interacting, processes. First, all networks show a reduced excitability in the range of a few seconds after an evoked network-wide burst. Second, a slow and oscillatory change of network excitability dramatically affected the number of spikes, which could be evoked during a given stimulation experiment. The slow change of the excitability spontaneously emerged in cultures with intact, but not in cultures with absent fast GABAergic synaptic transmission.

### Direct Responses

It is assumed that direct neuronal responses mainly reflecting antidromically activated soma through stimulated (Wagenaar et al., 2004). Stimulation electrodes might, in turn, also excite soma. In this case spikes, the extracellular correlates for intracellular action potentials, are recorded from the axons. Even stimulation of and recording from the same axon is conceivable. These different possibilities cannot be distinguished clearly by the shapes of recorded spike waveforms or by microscopically inspection of the culture. A classification of single neurons (i.e., determining whether a recorded neuron is inhibitory or excitatory) on the basis of their extracellular recorded firing properties is also not feasible (Weir et al., 2014). These limitations impede the interpretation of experimental data and should be kept in mind in stimulation studies.

The present results indicate ranges of frequencies, which reliably can evoke a neuronal response and ranges, which are fairly ineffective (**Figures 1** and **2**). Direct neuronal responses were evoked with little latency and with very high reliability (near 100%) for low stimulation frequencies (≈1–10 Hz), which is in agreement with previous results (Jimbo et al., 2000; Wagenaar et al., 2004). At higher stimulation rates, however, the reliability strongly decreased. That is, the ratio of applied pulses to evoked responses was initially almost 100% and decreased to ≈3% when changing the pulse rate from 1 to 100 Hz. This low-pass behavior might, in addition to the passive membrane properties, be the result of calcium-activated potassium conductances as a consequence of repetitive action potential firing at the beginning of a pulse train (see **Figures 1A** and **2**). Spikes with smaller amplitudes, which occurred at higher stimulation rates, might also indicate an incomplete recovery of the sodium conductance from a previous action potential (Gal et al., 2010). The sodium conductance, then, might recover partially during ongoing stimulation, when single stimulation pulses fail to evoke an action potential. At higher stimulation rates, however, stimulation pulses might interrupt the recovery, which would account for the inefficiency of high stimulation frequencies in entraining the neurons. However, we cannot exclude the possibility that the stimulation pulses occluded a number of spikes in our data.

Sustained electrical stimulation can effectively suppress synchronized burst activity, which has potential applications for the treatment of central nervous system disorders (Wagenaar et al., 2005). Assuming that extracellular pulses interfere with sodium conductances and/or calcium activated conductances of a neuron, which fires at moderate or high rates without electrical stimulation, implies that stimulation pulses can reduce the average firing rate, for example, by keeping the sodium conductance inactivated. This interference, then, could be the basis of a potent mechanism on the cellular level, which, together with synaptic mechanisms, is accountable for burst suppression by means of extracellular electrical stimulation. Whereas on the network level, burst suppression could be attributed to a depletion of activity depended synaptic reserves (Baltz et al., 2011).

### Dependency of the Evoked Responses on the Stimulation Frequency

Spikes that were evoked within the first milliseconds post stimulus reflect responses from the directly stimulated neuronal tissue (see above) and the earliest responses of synaptically activated neurons (Wagenaar et al., 2004). The developmental increase of the number of early spikes, therefore, most likely is attributable to the ongoing growth of the neurites and the maturation of synaptic connections.

The strong increase of the overall evoked network activity with development is related to the emerging burst response which preferably occurred at low stimulation rates (**Figure 4**). This late response showed marked differences in the temporal evolution of the firing rate for cultures with and without functional fast GABAergic synaptic transmission. Evoked reverberating bursts in cultures with blocked GABAergic synaptic transmission occurred in an all-or-none manner and often had multiple discharges in cultures older than 3 weeks (**Figure 4**). On the contrary, the firing frequency decayed after an initial discharge in unblocked controls, possibly as a result of the hyperpolarizing or shunting GABA action (Baltz et al., 2010). The prolonged discharges in cultures with blocked GABA_A_Rs may lead to a deeper synaptic depression, which could potentially lead to longer refractory periods (see below).

### Network Refractoriness

In line with previous data (Opitz et al., 2002; Baljon et al., 2009), double-pulse experiments showed second-long periods of low excitability after evoked reverberating discharges. In contrast to cultures with blocked GABA_A_Rs, in which the recovery occurred between 2 and 15 s, a gradual recovery of the excitability was found on a shorter time scale, starting at about 10–100 ms after a previous stimulation pulse in unblocked cultures. Moreover, in control cultures, as well in cultures with blocked fast GABAergic synaptic transmission, the second pulse did not evoke a significant amount of activity at inter-pulse intervals in the range of a few milliseconds, which might relate to axon refractoriness.

In juvenile rat cortex, synapses show an augmented synaptic depression (Reyes and Sakmann, 1999). The reduced excitability may, therefore, be mainly the result of the exhaustion of activity-dependent reserves, such as transmitter depletion in the readily releasable pool of the synapses, which leads to synaptic depression (Zucker and Regehr, 2002; Baltz et al., 2011).

A synaptic depression may be generally weaker in cultures with intact GABAergic transmission due to inhibition of neuronal activity. This would be in line with the observation that strongest reverberating burst responses in cultures with functional fast GABAergic synaptic transmission were considerably weaker compared with reverberating responses that occurred in an all-or-none manner in cultures with blocked GABA_A_Rs. Hence, a stronger depression of more synapses would be expected after stronger reverberating bursts in blocked cultures, leading to longer recovery phases and, thus, longer periods of low excitability.

Double-pulse experiments showed that, during a limited range of inter-pulse intervals, around 10 ms, the responses to the second pulse were enhanced in control cultures compared with networks where fast GABAergic transmission was blocked. In terms of synaptic depression, these additional spikes might reflect the recruitment of synapses, which were, due to fast synaptic inhibition, less affected by synaptic depression that was induced by the first pulse. As a result of the activity evoked by the first pulse, synaptic depression could gradually increase in time, which might explain the partial and transient reduction of the excitability for inter-pulse intervals between 20 and100 ms in cultures with intact GABAergic synaptic transmission (**Figure 6C**).

### Slow Periodic Changes of the Network Excitability

Prolonged low-frequency stimulation of ≈3–5 week old cultures with intact fast GABAergic synaptic transmission revealed slow and systematic GABA-dependent fluctuations of the network excitability. An observation which was not described in depth previously, but apparently is present under a variety of experimental conditions [see for example Figure 5 in Eytan et al. (2003); Figure 1D in Wagenaar et al. (2006b); Figure 1D in Chiappalone et al. (2008); Figure 2B in Shahaf et al. (2008)].

The slow changes of the excitability closely matched temporal fluctuations of the spontaneous network activity (**Figure 10**). Furthermore, most of the activity in the network was triggered by electrical pulses (**Figures 8** and **10**). These data strongly suggest that the amount of electrically evoked activity is governed by an underlying oscillation of the network excitability, which is largely independent of extracellular stimulation itself.

The impact of such slow and *oscillatory* changes on the experimental outcome might have been underestimated in recent studies, potentially resulting in the conflicting evidence concerning the amount of plastic changes that can be induced by means of extracellular pulses in dense cortical cultures with strongly synchronized burst activity (Jimbo et al., 1998; Jimbo et al., 1999; Shahaf and Marom, 2001; Eytan et al., 2003; Wagenaar et al., 2006b; Chiappalone et al., 2008; Dranias et al., 2013).

Periods of higher excitability are potentially the result of more depolarized membrane potentials in a significant fraction of the neurons. Interestingly, recurring phases of high and low network activity are reminiscent of slow potential shifts in juvenile hippocampal slices (Jensen and Yaari, 1997). The dynamics of the slow membrane potential changes appear to be a result of feedback interactions between neuronal discharges and the extracellular potassium concentration (Jensen and Yaari, 1997; Frohlich et al., 2006, 2008). Because the neuron specific potassium chloride co-transporter (Blaesse et al., 2009) provides a key link between the extracellular potassium concentration and fast GABAergic synaptic transmission (Blaesse et al., 2009; Viitanen et al., 2010), it is interesting to note that the occurrence of the slow oscillatory changes in the spontaneous network activity during the development is paralleled by the GABA shift in cortical cultures (Baltz et al., 2010) and that the GABA shift is associated with the developmental up-regulation of KCC2 (Rivera et al., 1999; Ben-Ari, 2002; Yamada et al., 2004; Ben-Ari et al., 2007). Indeed, KCC2 is strongly up-regulated in the present culture model during the second week *in vitro* (Westerholz et al., 2013). It might, therefore, be tempting to speculate that slow GABA_A_R-dependent potassium dynamics (Kaila et al., 1997; Rivera et al., 2005; Blaesse et al., 2009; Viitanen et al., 2010) are involved in the slow oscillating changes of the network excitability in cultured cortical networks.

GABAergic synaptic transmission in cortical cultures seems to be provided to a significant extend by the population of early post-mitotic large GABAergic cells, which have distinctive morphological and molecular features (de Lima and Voigt, 1997; Voigt et al., 2001; de Lima et al., 2004; Baltz et al., 2010). With respect to potassium dynamics, this might be of potential interest regarding the ‘potassium accumulation hypothesis’ in models of epilepsy [for review see Frohlich et al. (2008)], because distinct subpopulations of GABAergic neurons could provide the strong GABAergic drive that might preferentially induce potassium-mediated epileptic bursts in more structured networks.

The distribution of evoked spikes in response to electrical stimulation can convey a near linear relationship over several time scales in logarithmic space (**Figure 8C**). This power-law-like behavior is, however, the result of the very slow GABA-dependent oscillatory changes of the network excitability. Even a very clear exponential relationship between the number of evoked spikes (or the duration of the evoked response) and the frequency of occurrence would, therefore, give no information about the underlying mechanisms [e.g., a “critical branching process,” Pasquale et al. (2008)] or the network topology [e.g., scale free, Eytan and Marom (2006), Pasquale et al. (2008)].

### Putative Implications of Absent GABA_A_ergic Synaptic Transmission

In the absence of GABA_A_R mediated synaptic transmission it would be expected that homeostatic mechanisms counteract lacking GABA_A_ergic inhibition to prevent over-excitation. Such homeostatic modifications on the synaptic or cellular level (Turrigiano and Nelson, 2004; Turrigiano, 2007) in networks with lacking GABA_A_ergic transmission, if functionally relevant, could lead to an overall reduced activity in comparison to networks with intact inhibition. However, our data do not implicate a general trend in this context. Networks lacking GABA_A_ergic transmission failed to down regulate excitatory drive over the course of development to prevent the stereotyped hyper-synchronous activity. Thus, the limits of homeostatic regulation might be reached if the local circuitry is abnormally constructed on the cellular (Baltz et al., 2010) or synaptic level, in particular in the case of impaired GABA_A_R mediated transmission. As the excitatory role of GABAergic synaptic transmission seems to dominate in immature networks [for review see Ben-Ari et al. (2007)], and GABA becomes inhibitory roughly during the third week *in vitro* in cultured networks (Baltz et al., 2010) structural changes might, therefore, develop in different directions during the course of development in networks with absent GABA_A_ergic drive. Whether the pure absence of the GABAergic drive or structural changes dominate the response properties during different developmental stages remains to be investigated.

### Implications for Future Stimulation Studies

For pharmacologically isolated neurons we showed ranges of frequencies, which reliable evoke a neuronal response, and ranges, which were less effective. This should be taken into account, as higher stimulation rates do not necessarily imply higher neuronal firing rates. As discussed above, even a significant depression of neuronal activity must be considered. On the network level, the amount and phenomenology of the electrically evoked activity of cortical cultures *in vitro* depends on the developmental stage and is modulated by an activity-dependent process and very slow GABA-dependent periodic changes of the network excitability. Both have to be taken into account when investigating evoked activity of cortical networks *in vitro*, since for example, very slow spontaneous changes of the excitability may lead to spurious findings when trying to induce plastic changes by means of extracellular pulses. Furthermore, the impact of absent fast GABAergic synaptic transmission on the evoked responses was shown throughout the development of cortical networks *in vitro*, which may help to clarify whether or not fast GABAergic synaptic transmission is involved in future stimulation experiments. Since the observed response properties can be explained in part by general cellular properties or by the bursting nature of the network activity with an underlying synaptic depression (Baltz et al., 2011), the latter being hallmark feature of young rat cortex (Feller, 1999; Reyes and Sakmann, 1999; Ben-Ari, 2001), some of the present results should qualitatively extend to more structured networks.

## Author Contributions

TB, TV conceived and designed the experiments; TV prepared cell cultures, TB performed the stimulation experiments; TB, TV analyzed the data and wrote the paper.

## Conflict of Interest Statement

The authors declare that the research was conducted in the absence of any commercial or financial relationships that could be construed as a potential conflict of interest.

## Abbreviations

AP5: (2R)-amino-5-phosphonovaleric acid
CNQX: 6-cyano-7-nitroquinoxaline-2,3-dione
C-Pulse: conditioning pulse
DIV: days *in vitro*
GABA_A_R: GABA receptor type A
ISI: inter spike interval
KCC2: potassium chloride cotransporter 2
MEA: multielectrode arrays
SNR: single-to-noise ratio
T-Pulse: test pulse.
